# Donor–π–donor type hole transporting materials: marked π-bridge effects on optoelectronic properties, solid-state structure, and perovskite solar cell efficiency†
†Electronic supplementary information (ESI) available. CCDC 1446682–1446684. For ESI and crystallographic data in CIF or other electronic format see DOI: 10.1039/c6sc01478j


**DOI:** 10.1039/c6sc01478j

**Published:** 2016-05-24

**Authors:** S. Paek, I. Zimmermann, P. Gao, P. Gratia, K. Rakstys, G. Grancini, Mohammad Khaja Nazeeruddin, Malik Abdul Rub, Samia A. Kosa, Khalid A. Alamry, Abdullah M. Asiri

**Affiliations:** a Group for Molecular Engineering of Functional Materials , Ecole Polytechnique Federale de Lausanne Valais Wallis , Rue de l'Indutrie 17 , 1950 Sion , Valais , Switzerland . Email: mdkhaja.nazeeruddin@epfl.ch ; Email: peng.gao@epfl.ch; b Center of Excellence for Advanced Materials Research (CEAMR) , King Abdulaziz, University , Jeddah , Saudi Arabia

## Abstract

Donor–π-bridge–donor type oligomers (D–π–D) have been studied intensively as active materials for organic optoelectronic devices. In this study, we introduce three new D–π–D type organic semiconductors incorporating thiophene or thienothiophene with two electron-rich TPA units, which can be easily synthesized from commercially available materials. A thorough comparison of their optoelectronic and structural properties was conducted, revealing the strong influence of the extent of longitudinal π-bridge conjugation on both the solid structure of the organic semiconductive materials and their photovoltaic performance when applied as hole transporting materials (HTM) in perovskite solar cells. Single-crystal measurements and time-resolved photoluminescence (TRPL) studies indicate that these coplanar donor–π–donor type HTMs could be promising alternatives to state-of-the-art spiro-OMeTAD, due to the multiple intermolecular short contacts as charge transporting channels and efficient charge extraction properties from the perovskite layer. The optimized devices with **PEH-9** exhibited an impressive PCE of 16.9% under standard global AM 1.5 illumination with minimized hysteretic behaviour, which is comparable to that of devices using spiro-OMeTAD under similar conditions. Ambient stability after 400 h revealed that 93% of the energy conversion efficiency was retained for **PEH-9**, indicating that the devices had good long-term stability.

## Abstract 2

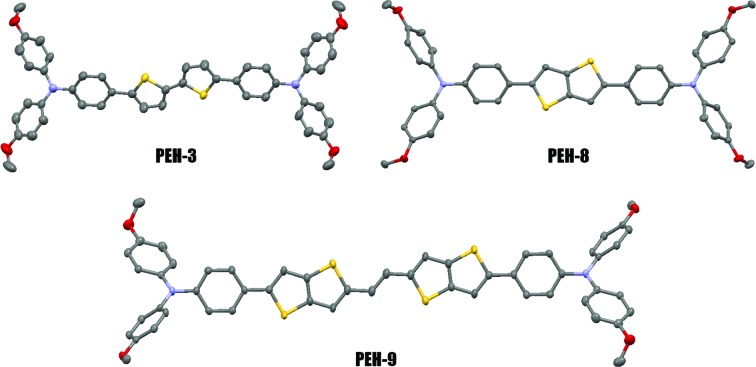
Donor–π-bridge–donor type oligomers (D–π–D) have been studied intensively as active materials for organic optoelectronic devices.

## Introduction

Organometal halide perovskite solar cells (PSCs) exhibiting high power conversion efficiencies (PCEs) may provide inexpensive, renewable sources of solar electricity *via* low-cost materials and fabrication techniques.^1^–^3^ PCEs of PSCs have been quickly increased from 3.8 to 22.1% as certified by the National Renewable Energy Laboratory (NREL)^4^ due to their intrinsic advantages such as broad absorption in the visible region,^5^ high absorption coefficients,^6^ high charge carrier mobility^7^ and long diffusion length.^8^ In such devices, the photoactive layer normally consists of a pure/blended polycrystalline layer of perovskite semiconductor [APbX_3_, A = MAI, FAI, Cs; X = Cl, Br, I] that is imbedded between a layer of electron transporting material (ETM) and a hole transporting material (HTM).^2^ An attractive approach to push PSCs to industry and market, besides developing unconventional device structures^9^ and more complicated perovskite compositions,^10^ is to explore new contact/interfacial materials, particularly HTMs.^11^ HTMs play an important role in determining the photovoltaic performance and long-term stability of the perovskite solar cells. Among the many HTMs developed, 2,2′,7,7′-tetrakis(*N*,*N*-di-*p*-methoxyphenylamine)-9,9′-spirobifluorene (spiro-OMeTAD) is by far the most studied and used molecular *p*-type HTM with a recently reported PCE of 20.8%.^12^ However, spiro-OMeTAD is very expensive owing to the need for sublimation for purification. In this regard, the development of cost-effective and efficient HTMs remains a problem.

Recently, impressive photovoltaic performance has been achieved using molecular HTMs, such as thiophene derivatives,^13^,^14^ 3,4-ethylenedioxythiophene derivatives,^15^–^17^ spiro-OMeTAD derivatives,^18^,^19^ truxene-based derivatives,^20^ carbazole derivatives,^21^*etc.*^22^ Their characterization provides fundamental information on how molecular modifications affect PCE by altering arylamine-substitution, π-system size, steric geometry, and carrier mobility. However, the interesting question of how π-bridge conjugation variations in HTM materials, which marginally affect solution phase molecular properties, influence the photovoltaic performance of the PSC remains unanswered.

**Fig. 1:**
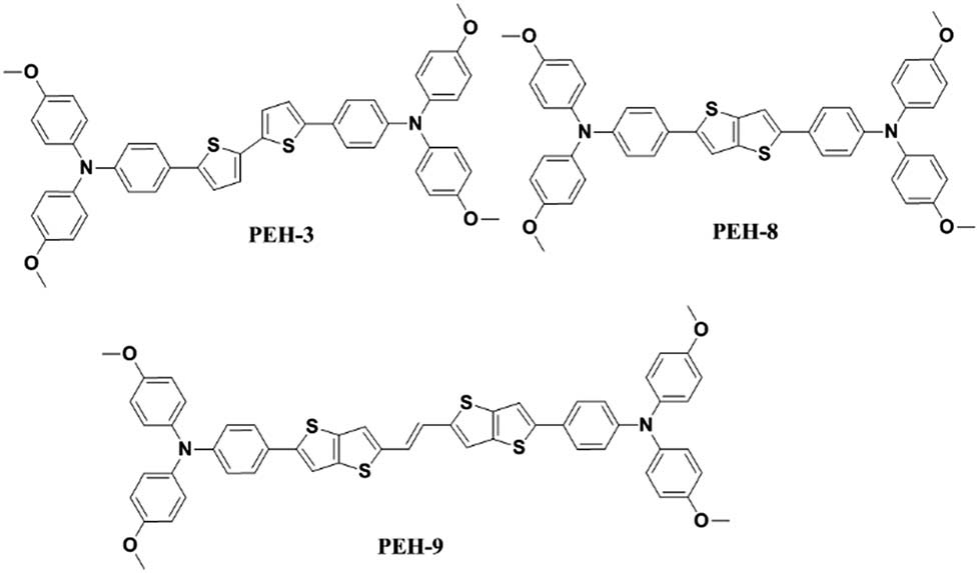
Chemical structures of **PEH-3**, **PEH-8** and **PEH-9**.

Oligothiophenes form an important class of organic semiconductors that are widely used in the fields of organic field effect transistors (OFETs) and organic photovoltaics (OPVs).^23^ They are relatively easily synthesized and have been shown to be viable models for predicting and understanding the electronic structures of more complicated organic π-systems. Herein, we report the facile synthesis and characterization of three new donor–π–donor type HTMs for PSCs, denoted as **PEH-3**, **PEH-8** and **PEH-9**, by incorporating simple thiophene or thienothiophene with an ethylene unit as the π-bridge (Fig. 1). The new compounds were characterized by conventional spectroscopic/analytical/X-ray diffraction methodologies. The remarkable solid structure differences were delineated in the performance of the PSCs. **PEH-9** with more abundant intermolecular atomic contacts shows an excellent overall efficiency close to 17%. This study underscores the value of increased charge transporting channels after longitudinal conjugation extension by thiophene substitution, and complements recent work by Ganesan *et al.*^16^ and Li *et al.*^14^ on quasi-spiro ethylenedioxythiophene and swivel-cruciform thiophene based HTMs, respectively, which emphasized lateral expansion of molecular structure.

## Results and discussion

### Synthesis

The three new HTMs were efficiently synthesized by the stepwise synthetic protocol illustrated in Scheme S1.† Compound 1 was prepared according to reported procedures.^24^ The McMurry reaction^25^ of compound 1 with 1.2 equivalents of TiCl_4_ and then 2.4 equivalents of Zn powder produced **PEH-9**. The Suzuki coupling reactions of 5,5′-dibromo-2,2′-bithiophene and 2,5-dibromothieno[3,2-*b*]thiophene with 2.5 equivalents of 4-methoxy-*N*-(4-methoxyphenyl)-*N*-(4-(4,4,5,5-tetramethyl-1,3,2-dioxaborolan-2-yl)phenyl)aniline in THF/H_2_O produced **PEH-3** and **PEH-8**, respectively. The chemical structures of the synthesized products were verified by ^1^H and ^13^C NMR spectroscopy (see ESI†) and MALDI-TOF mass spectrometry.

#### (*E*)-4,4′-(5,5′-(Ethene-1,2-diyl)bis(thieno[3,2-*b*]thiophene-5,2-diyl))bis(*N*,*N*-bis(4-methoxyphenyl)aniline) (**PEH-9**)

Titanium(iv) chloride (0.083 ml, 0.76 mmol) was added dropwise to compound 1 (0.3 g, 0.64 mmol) dissolved in THF over a period of 30 min at –18 °C. After stirring at this temperature for 30 min, zinc powder (0.1 g, 1.5 mmol) was added in small portions over a period of 30 min. The mixture was stirred at –18 °C for 30 min and refluxed overnight. The reaction was quenched with ice water and methylene chloride was added, then collected by filtration. The solution was extracted and dried with MgSO_4_. The product was purified by column chromatography (EA : Hx = 1 : 5). MS: *m*/*z* 910.128 [M^+^]. ^1^H NMR (CDCl_3_): *δ* 7.33 (d, 4H, ^3^*J* = 8 Hz), 7.21 (s, 2H), 7.05 (s, 2H), 7.01 (d, 8H, ^3^*J* = 8 Hz), 6.95 (s, 2H), 6.84 (d, 4H, ^3^*J* = 8 Hz), 6.78 (d, 8H, ^3^*J* = 8 Hz), 3.75 (s, 12H). ^13^C{^1^H} NMR (CDCl_3_): *δ* 156.1, 148.5, 147.0, 143.3, 140.4, 139.0, 137.8, 126.8, 126.4, 126.3, 121.6, 120.2, 118.7, 114.7, 113.9, 55.5. Anal. calc. for C_54_H_42_N_2_O_4_S_4_: C, 71.18; H, 4.65; N, 3.07. Found: C, 71.15; H, 4.66; N, 3.01.

#### 4,4′-(Thieno[3,2-*b*]thiophene-2,5-diyl)bis(*N*,*N*-bis(4-methoxyphenyl)aniline) (**PEH-8**)

Under a nitrogen atmosphere, a mixture of 4-methoxy-*N*-(4-methoxyphenyl)-*N*-(4-(4,4,5,5-tetramethyl-1,3,2-dioxaborolan-2-yl)phenyl)aniline (1.3 g, 3 mmol), 2,5-dibromothieno[3,2-*b*]thiophene (0.3 g, 1 mmol), Pd(PPh_3_)_4_ (0.11 g, 0.1 mmol), K_2_CO_3_ (7.4 g, 5 mmol) in degassed water (20 ml) and dry THF (40 ml) was refluxed for 2 days. After cooling to room temperature, the solution was extracted with dichloromethane and dried with MgSO_4_. The solvent was removed *in vacuo*. The product was purified by column chromatography (DCM : Hx = 1 : 1). MS: *m*/*z* 745 [M^+^]. ^1^H NMR (CDCl_3_): *δ* 7.44 (d, 4H, ^3^*J* = 8 Hz), 7.32 (s, 2H), 7.10 (d, 8H, ^3^*J* = 8 Hz), 6.94 (d, 4H, ^3^*J* = 8 Hz), 6.87 (d, 8H, ^3^*J* = 8 Hz), 3.85 (s, 12H). ^13^C{^1^H} NMR (CDCl_3_): *δ* 156.0, 148.3, 145.4, 140.5, 138.4, 126.8, 126.7, 126.3, 120.4, 114.7, 113.8, 55.5. Anal. calc. for C_46_H_38_N_2_O_4_S_2_: C, 73.97; H, 5.13; N, 3.75. Found: C, 73.99; H, 5.11; N, 3.80.

#### 4,4′-([2,2′-Bithiophene]-5,5′-diyl)bis(*N*,*N*-bis(4-methoxyphenyl)aniline) (**PEH-3**)

**PEH-3** was synthesized by a similar procedure to **PEH-8**, except that 5,5′-dibromo-2,2′-bithiophene was used in place of 2,5-dibromothieno[3,2-*b*]thiophene. The product was purified by column chromatography (DCM : Hx = 1 : 1). MS: *m*/*z* 771 [M^+^]. ^1^H NMR (CDCl_3_): *δ* 7.41 (d, 4H, ^3^*J* = 8 Hz), 7.10 (d, 12H, ^3^*J* = 8 Hz), 6.94 (d, 4H, ^3^*J* = 8 Hz), 6.87 (d, 8H, ^3^*J* = 8 Hz), 3.83 (s, 12H). ^13^C{^1^H} NMR (CDCl_3_): *δ* 156.0, 148.2, 143.1, 140.6, 135.5, 126.6, 126.2, 124.1, 122.3, 120.4, 114.7, 55.5. Anal. calc. for C_48_H_40_N_2_O_4_S_2_: C, 74.58; H, 5.22; N, 3.62. Found: C, 74.55; H, 5.21; N, 3.64.

### Optical and thermal properties

**Fig. 2:**
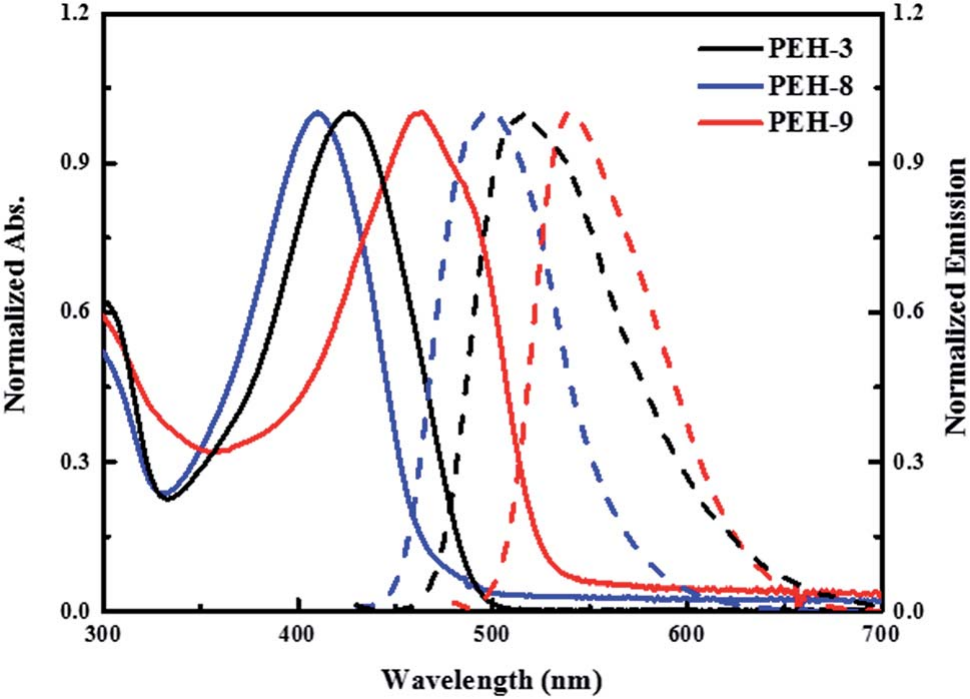
UV-vis absorption (solid line) and emission (dashed line) spectra of **PEH-3** (black), **PEH-8** (blue) and **PEH-9** (red) in dichloromethane.

The UV-vis absorption and emission spectra of **PEH-3**, **PEH-8** and **PEH-9** in dichloromethane are shown in Fig. 2. **PEH-3** shows an absorption maximum at 426 nm, which is due to the π–π* transition of the conjugated system. Under the same conditions, **PEH-8** incorporating thieno[3,2-*b*]thiophene exhibits a slightly blue shifted absorption band at 410 nm due to the decreased number of double bonds, whereas **PEH-9** with increased conjugation shows an absorption maximum at 463 nm. The spectrum of **PEH-9** is red shifted as compared to **PEH-3** and **PEH-8** due to increased conjugation length with the addition of more heteroaromatic rings. The emission spectra show that **PEH-3**, **PEH-8** and **PEH-9** have large Stokes shifts of about 90, 88 and 78 nm, respectively, which suggests significant changes in the geometrical configuration of the molecules upon excitation. The optical band gap (*E*_0–0_) of **PEH-3**, **PEH-8** and **PEH-9** is calculated (2.60, 2.71 and 2.41 eV) from the intersection of absorption and emission spectra. To determine the thermal properties of the three new HTMs, we carried out thermogravimetric analysis (TGA) (see Fig. S1 in ESI†). Although **PEH-9** with increased conjugation length is less stable compared with **PEH-8** and **PEH-3**, all the HTMs started to decompose at a temperature around 400 °C.

### Electrochemical properties, DFT calculations and hole mobility

**Fig. 3:**
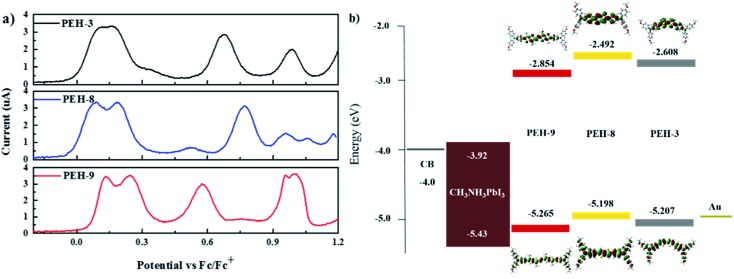
(a) Differential pulse voltammetry (DPV) of the three compounds with 0.1 M tetrabutylammonium hexafluorophosphate in CH_2_Cl_2_ at a scan speed of 50 mV s^–1^, potentials *vs.* Fc/Fc^+^; (b) energy level diagram of each component in a hybrid solar cell calculated based on DPV measurements with isodensity surface plots of **PEH-3**, **PEH-8** and **PEH-9** as calculated by time dependent-density functional theory (TD-DFT) using the B3LYP functional/6-31G* basis set.

The electrochemical behaviour of **PEH-3**, **PEH-8** and **PEH-9** was investigated by cyclic voltammetry (CV) as shown in Fig. S2† and differential pulse voltammetry (DPV) as shown in Fig. 3a. The values are summarized in Table 1. The three new HTMs show two reversible redox processes in the low potential region at around 0.15 V and 0.25 V, respectively, indicating that the two electrons are removed successively from the two donor triphenyl amine (TPA) units and a higher potential is needed to transform HTM^+^˙ into HTM^2+^˙. The small difference in the two oxidation peaks of **PEH-3** indicated that the two electrons are removed almost simultaneously from the two TPA units to form a dication in a similar region. However, in the case of the **PEH-8** and **PEH-9** HTMs, the two electrons are removed sequentially. The splitting of the oxidation of the two TPA units into two steps suggests effective charge delocalization through the π-bridge between the two TPA branches. The redox processes of the three HTMs are completely reversible, with **PEH-9** having the lowest oxidation potential and band gap due to increased conjugation length. The HOMO energy levels were calculated from the CV and DPV data with assumptions following the procedure previously described.^26^ The HOMO levels calculated from the oxidation potential maxima in DPV are –5.207, –5.198 and –5.265 eV for **PEH-3**, **PEH-8** and **PEH-9**, respectively. As the HOMO energy level of CH_3_NH_3_PbI_3_ is –5.43 eV,^27^ the three new HTMs should have an efficient hole extraction capability. LUMO energy levels were calculated according to *E*_LUMO_ = *E*_HOMO_ – *E*_0–0_ to be –2.608, –2.492 and –2.854 eV, respectively, which will allow sufficient offset of the conduction band of perovskite to not only block the electrons from perovskite, but also to ensure a cascade of electron transfer at the interface when the HTMs are excited (Fig. 3b).

**Table 1:** Optical, electrochemical, thermal and mobility parameters of the compounds

Compounds	*λ*_abs_ ^*a*^/nm (*ε*/M^–1^ cm^–1^)	*E*_0–0_ ^*b*^ (eV)	*E* ox 0 ^*c*^ (V)	*E*_HOMO_ ^*d*^ (eV)	*E*_LUMO_ ^*e*^ (eV)	*T*_deg_ ^*f*^ (°C)	*μ*, cm^2^ V^–1^ s^–1^
**PEH-3**	426 (45 200)	2.60	0.117	–5.207	–2.608	405	3.72 × 10^–5^
**PEH-8**	410 (55 200)	2.71	0.068	–5.198	–2.492	410	4.05 × 10^–5^
**PEH-9**	463 (86 000)	2.41	0.135	–5.265	–2.854	395	3.64 × 10^–6^

^*a*^Absorption spectra were measured in CH_2_Cl_2_ solution.

^*b*^ *E*_0–0_ was from the absorption and emission cross peak in dichloromethane solution.

^*c*^Redox potentials of the compounds were measured in CH_2_Cl_2_ with 0.1 M (*n*-C_4_H_9_)_4_NPF_6_ with a scan rate of 50 mV s^–1^ (*vs.* Fc/Fc^+^).

^*d*^The energy of the highest occupied molecular orbital (*E*_HOMO_) was calculated as *E*ox0 (V) *vs.* Fc/Fc^+^ + 0.69 *vs.* NHE + 4.44 *vs.* vacuum.

^*e*^The energy of the lowest unoccupied molecular orbital (*E*_LUMO_) was calculated according to *E*_0–0_ – *E*_ox_.

^*f*^Degradation temperature observed from TGA 5% weight loss at 10 °C min^–1^, N_2_ atmosphere.

Calculations using time dependent-density functional theory (TD-DFT) were used to investigate the electronic properties of the HTMs. As shown in Fig. 3b, the orbital density of the HOMO of the three new HTMs is located over the entire molecule, whereas the orbital density of the LUMO is predominantly located on the bridge group, since the (thieno)thiophene unit has less electron-donating strength than the TPA unit. All molecules have a similar orbital density due to their similar donor–π–donor structure.

The hole drift mobility of the HTM layers was measured on OFET substrates (Fig. S3†). The mobility (*μ*) values of oxidized **PEH-3**, **PEH-8** and **PEH-9** were determined to be 3.72 × 10^–5^ cm^2^ V^–1^ s^–1^, 4.05 × 10^–5^ cm^2^ V^–1^ s^–1^ and 3.64 × 10^–6^ cm^2^ V^–1^ s^–1^, respectively, which are lower than that of spiro-OMeTAD (*μ* = 5 × 10^–5^ cm^2^ V^–1^ s^–1^). All electrical properties are summarized in Table 1.

### Single-crystal packing and analysis

**Fig. 4:**
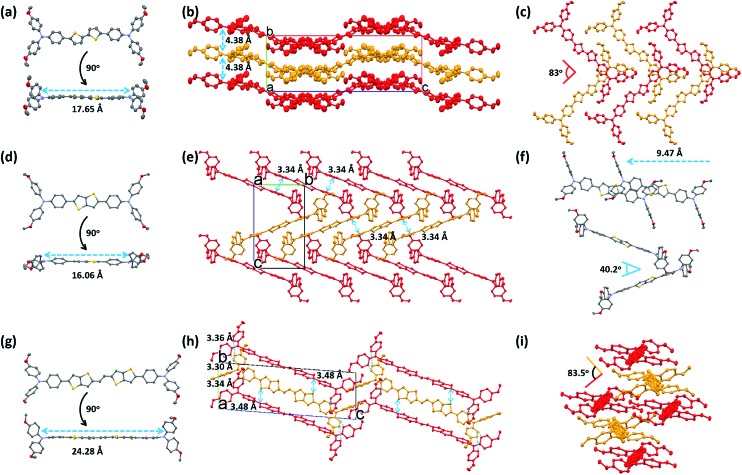
Crystal structures of the three TPA-substituted oligomer derivatives at 273 K and 180 K. Thermal ellipsoids are set at 50% probability. The red-, yellow-, purple-, and gray-colored atoms represent O, S, N, and C, respectively. All of the hydrogen atoms have been omitted for clarity. (a, d, g) ORTEP structures of the three HTM derivatives from both face and edge perspectives: (a) **PEH-3**; (d) **PEH-8**; (g) **PEH-9**. (b, e, h) Crystal packing diagrams of the single crystals of the three HTM derivatives: (b) **PEH-3** (*Pbca* (61) space group, CH···π distance of 2.77 Å, interlayer distance of 4.38 Å); (e) **PEH-8** (*P*121/*n*1 (14) space group, π···π distance of 3.34 Å); (h) **PEH-9** (*P*1[combining macron] (2) space group, π···π distances of 3.30, 3.34 and 3.36 Å, S···π distances of 3.48 Å) (red and yellow colors are used to highlight the different layers). (c, f, i) Molecular packing arrangements of the three HTM derivatives from different stacking directions: (c) **PEH-3** (top view of slipped 2D stacking layers, intersection angle of longitudinal direction is 83°); (f) **PEH-8** (longitudinal shift is 9.47 Å and intersection angle of longitudinal direction is 40.2°); (i) **PEH-9** (edge to face alternating stacking with intersection angle of transverse direction 83.5°).

The differences found in the electronic properties of semi-conducting materials can be determined from the crystal structures. Single crystals of these new molecules were obtained by the slow solvent evaporation method. The detailed crystallographic data are summarized in Tables 2 and S1.† Comparison between the crystal structures of these compounds with different π-bridges provides insight into how the conjugation length of the thiophene based bridge affects molecular structure and packing characteristics. The overall geometry of the molecules is substantially different from that of Spiro-OMeTAD.^16^ The conjugated bridges of the **PEH-3** and **PEH-9** exhibit an all-anti, fully planar geometry, with dihedral angles between TPA and the mean plane of the rings <3°. In contrast, the thienothiophene bridging **PEH-8** exhibits a much higher dihedral angle of 22° (Fig. 4a, d and g and S4†). The molecular packing of these new HTMs with longer and shorter bridges is radically different. **PEH-3** with a medium bridge length of 17.65 Å showed layer-by-layer stacking geometry. From the *a* axis of the single crystal of **PEH-3**, each layer shows a waved conformation within which the interlaced **PEH-3** molecules interact with each other *via* eight CH···π hydrogen bonds. The bulky TPA moieties tend to flock together to form the ridge and valley. Like a familiar “herringbone” (HB) motif, the intersection angle between two adjacent molecules is 83° (Fig. 4b and c). Along the *b* axis, these 2D layers stack through an abundance of CH···π hydrogen bonds to form the crystal. The average distance between each layer is measured to be 4.38 Å, which is too large to induce π–π interactions. No co-facial overlap is observed, which is quite unexpected in view of the highly planar geometry in the π-bridge.

**Table 2:** Crystallographic data for the three triphenylamine-substituted oligomer derivatives

	**PEH-3**	**PEH-8**	**PEH-9**
Crystal system	Orthorhombic	Monoclinic	Triclinic
Space group	*Pbca* (61)	*P*121/*n*1 (14)	*P*1[combining macron] (2)
Calculated density (g cm^–3^)	1.239	1.280	1.291
Short contact distance (Å)	2.77 (CH···π)	3.34 (π···π)	3.48 (S···π)
		2.96 (CH···π)	3.30 (π···π)
		2.50 (CH···O)	3.34 (π···π)
		2.70 (CH···π)	3.36 (π···π)
			2.73–2.84 (CH···π)

Thanks to the small π–π distances of 3.34 Å, completely different packing is observed in the single crystal of **PEH-8**. The π–π interactions are measured between each **PEH-8** molecule and two adjacent molecules with the same longitudinal orientation (high longitudinal shift of 9.47 Å) (Fig. 4f). Considering the length of the central π-bridge is merely 16.06 Å, only a small part of each **PEH-8** molecular skeleton overlaps with the adjacent molecules. The slipped molecules arranged in a 1D stacking structure forming half of the “herringbone” motif. Each half of the “herringbone” motif forms one layer of the crystal as indicated by the red and yellow colors in Fig. 4e. Unlike the case of **PEH-3**, the “herringbone” structure is formed from the edge side of the long axis of the molecule. The intersection angle between two adjacent **PEH-8** molecules is 40.2° (Fig. 4f). The origin of this angle is probably due to interactions between the TPA moieties. Continuous regions of TPA and thienothiophene can be marked out along the *b* axis (Fig. S3b†). In addition to the π–π interactions and CH···π hydrogen bonds, CH···S and CH···π hydrogen bonds are also observed.

A more complex packing is observed for molecule **PEH-9**, which has the longest central π-bridge (24.28 Å). In this case, the high core planarity and the long conjugation length force the molecules to stack with the highest overlapping level. More importantly, the two thienothiophene units provide two additional S···π (3.48 Å) and CH···π interactions between each **PEH-9** molecule and two adjacent molecules, indicating the formation of a standard edge-to-face herringbone packing structure (with an angle of ∼83.5°) (Fig. 4h and i). At the same time, three minimal intermolecular (π···π) distances (3.30 Å, 3.34 Å, 3.36 Å) and even more CH···π interactions are measured in the TPA parts of the aggregated molecules. Similarly to **PEH-8**, continuous regions of TPA and the central π-bridge formed *via* self-assembly with much higher overlap (Fig. S4c†). From the single crystal study, we can see that out of the three new HTMs, **PEH-9** possesses the highest number/most types of intermolecular interactions.

### Perovskite solar cell evaluation

**Fig. 5:**
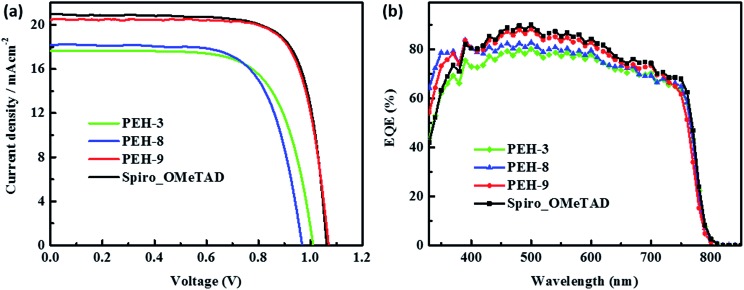
(a) Current (*J*)–voltage (*V*) curves of the solar cells with **PEH-3**, **PEH-8**, **PEH-9** and Spiro-OMeTAD control recorded from forward bias (FB) to short circuit conditions (SC) under AM 1.5 conditions (100 mW cm^–2^). (b) Incident photon-to-electron conversion efficiency (IPCE) as a function of wavelength for the PSCs in (a).

**Fig. 6:**
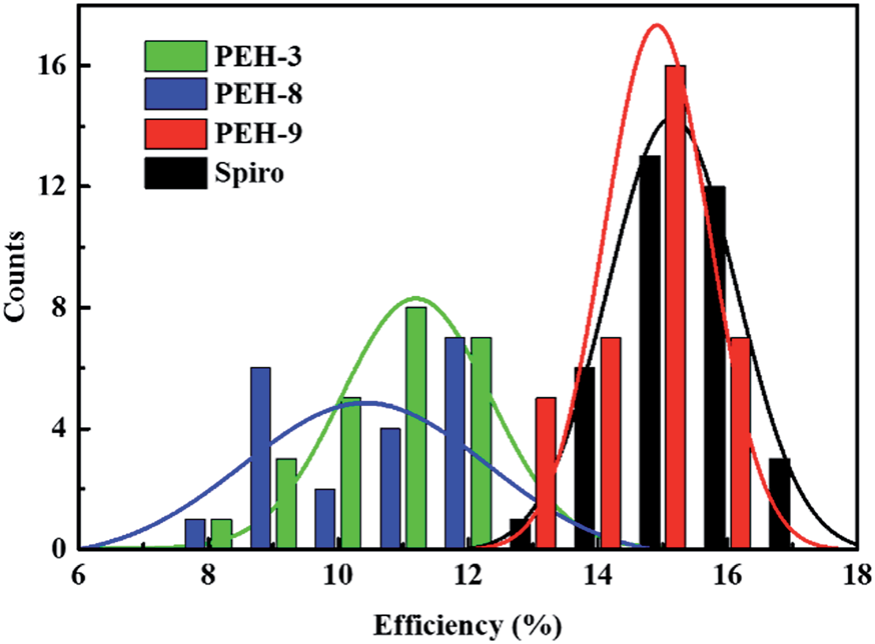
Statistical distribution of efficiencies of the perovskite solar cells with **PEH-3**, **PEH-8**, **PEH-9** and Spiro-OMeTAD. 30 devices were fabricated for each analysis. The distribution curve is fitted by the Gaussian function.

The perovskite solar cell devices with the new HTMs having a device configuration of FTO/TiO_2_ compact layer/TiO_2_ mesoporous/CH_3_NH_3_PbI_3_/HTM/Au were fabricated. Fig. 5a describe the current–voltage (*J*–*V*) curves (FB to SC) under standard global AM 1.5 illumination, fabricated using MAPbI_3_ perovskite as the absorber. It is known that *J–V* hysteresis between forward and reverse scans is typically observed in the *J*–*V* characteristic of PSCs, which makes estimation of the actual PCE ambiguous.^28^,^29^ To show the impact of the hysteresis on our device performances, we reported in Fig. S5† the *J*–*V* traces of PSCs based on these new HTMs collected by scanning the applied voltage at 0.01 V s^–1^ from forward bias (FB) to short circuit (SC) and the other way around. The solar-cell performance parameters extracted from both directions of the *J*–*V* curves are presented in Table 3. When **PEH-3** is tested, the PSC exhibits a PCE of 12.56% on the backward scan with a short-circuit photocurrent density (*J*_SC_) of 17.92 mA cm^–2^, open-circuit voltage (*V*_OC_) of 1008 mV, and fill factor (FF) of 0.695. For the forward scan, a slightly decreased PCE of 11.6% is observed leading to an average PCE of 12.08%. The average PCE decreases to 11.11% when **PEH-8** is used as the HTM layer due to more prominent hysteretic behaviour, which results in a much decreased PCE for the forward scan. The highest average PCE recorded is 16.6% for the PSC with **PEH-9** as the HTM layer, which displayed a *J*_SC_ of 20.42 mA cm^–2^, a *V*_OC_ of 1070 mV, and a fill factor of 76.5, and an overall efficiency of 16.9% for the reverse scan. The improved efficiency with **PEH-9** is due to the simultaneously increased *J*_SC_, *V*_OC_ and FF values associated with the increased intermolecular interaction and film quality (see Fig. S6†). We note that the average open circuit voltage (*V*_OC_) followed the trend of *V***PEH-9**OC > *V***PEH-3**OC > *V***PEH-8**OC. This can be rationalized by the similar trend in the *E*_HOMO_ levels of the new HTMs (Table 1). The *J*_SC_ values for these PSCs are quite similar and consistent with their respective incident photon-to-electron conversion efficiency (IPCE) spectra, which are shown in Fig. 5b. IPCE of all devices exceeds 70% in the broad spectral range from 400 to 700 nm, reaching a maximum of 80% at ∼500 nm. The integrated photocurrent density of **PEH-9** is 20.29 mA cm^–2^, which is in good agreement with the measured photocurrent density of 20.42 mA cm^–2^ at the standard solar AM 1.5G. Devices based on Spiro-OMeTAD were fabricated under the same conditions for comparison. It turned out that devices fabricated using **PEH-9** gave an overall performance (=16.6%) comparable to that of the devices based on Spiro-OMeTAD (=17.03%), indicating **PEH-9** as a new promising HTM for PSCs. The statistical data of the perovskite solar cells containing the three new HTMs and spiro-OMeTAD based on 30 identical devices with each HTM are shown in Fig. 6, giving average PCE values of 11.19%, 10.42%, 14.91% and 15.14%, respectively. These results exhibit a similar trend to the best performing devices. In addition, narrow efficiency distributions are observed for both **PEH-9** and spiro-OMeTAD. Finally, the operational stability of the new HTM-based perovskite solar cell was tested under a light intensity of 100 mW cm^–2^ and the data are shown in Fig. S8 (ESI†). The encapsulated cells showed diversified behavior over time depending on the HTM molecules used. Generally, the **PEH-9** and Spiro-OMeTAD showed comparable and better stability with ∼7% loss of PCE over 400 hours. On the contrary, **PEH-8** showed the most significant deterioration in PCE, mainly due to the decrease in *J*_SC_. The FF values of **PEH-3** and **PEH-8** decrease more prominently too. After 400 h in an ambient environment, the PCE of the device with **PEH-8** as the HTM retains only 60% of its initial value.

**Table 3:** Solar cell performance parameters

Materials	Scan direction	*J*_SC_, mA cm^–2^	*V*_OC_, mV	FF	PEC (%)
**PEH-3**	FB to SC	17.920	1008.0	69.50	12.560
	SC to FB	17.830	1003.0	64.90	11.600
	Average	17.875	1005.5	67.20	12.080
**PEH-8**	FB to SC	18.200	969.0	69.50	12.250
	SC to FB	18.100	864.0	58.90	9.970
	Average	18.150	916.5	64.20	11.110
**PEH-9**	FB to SC	20.420	1070.0	76.50	16.900
	SC to FB	20.450	1058.0	75.20	16.300
	Average	20.435	1064.0	75.85	16.600
Spiro	FB to SC	20.900	1074.0	77.70	17.420
	SC to FB	20.900	1063.0	75.50	16.650
	Average	20.900	1068.5	76.60	17.035

### Time-resolved photoluminescence (TRPL)

**Fig. 7:**
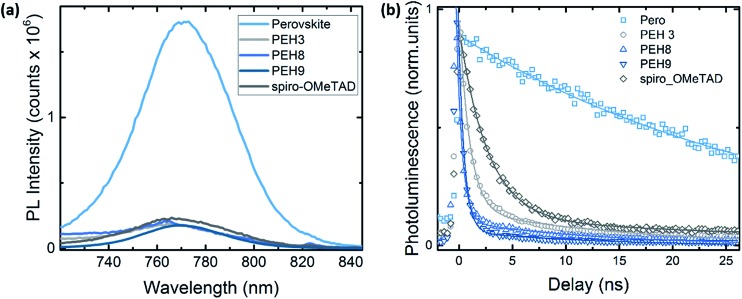
(a) Continuous wave (CW) photoluminescence spectra, excitation at 500 nm; (b) time resolved photoluminescence decay at 760 nm for the pristine perovskite film and the bilayer series. Excitation at *λ*_exc_ = 460 nm, excitation density of 1 nJ cm^–2^. All the samples have been encapsulated to prevent degradation or any oxygen/moisture induced effects. Solid lines represent the fitting curve resulting from exponential fitting in the form of *y* = *A*_1_ × exp(–*x*/*t*_1_) + *A*_2_ × exp(–*x*/*t*_2_). See Table S2† for the fitting parameters.

To better investigate the photoinduced processes behind the device operation, we monitored the photoluminescence dynamics of the different HTMs interfaced in a bilayer configuration with the perovskite. The continuous wave (CW) photoluminescence (PL) spectra of the perovskite and of the bilayers are reported in Fig. 7a. It is noted that all the bilayers show a dramatic quenching with respect to the pristine perovskite with similar intensity. The PL quenching can reasonably be attributed to the efficient hole transfer that happens at the perovskite/HTM interfaces. Fig. S7† shows the PL spectra of the same sample (**PEH-9** on perovskite) excited from both the perovskite side and the **PEH-9** side, from which we can estimate 90% quenching. Fig. 7b shows the comparison of the time resolved photoluminescence decay at 760 nm between the bare perovskite deposited on glass without any HTM on top and the series of bilayers, also including the reference Spiro-OMeTAD. The pristine perovskite film shows a long-living decay fitted with a monoexponential time constant above the time window (*t*_1_ = 31 ns, see Table S2†), in agreement with observations in the literature.^30^,^31^ On the other hand, the PL decay at the perovskite/spiro and perovskite/HTM exhibits a similar trend, consisting of a fast component of less than 1 ns and a long living one of around 10 ns. The quenching observed indicates an efficient hole transfer occurring within 1 ns, ultimately limited by our instrument resolution. Note that among the different HTMs, the bilayer with **PEH-9** shows a dominant contribution in relative weight in favour of the fast component (the amplitude *A*_1_ is approaching 98%). This behaviour might suggest that, among other interfacial processes, the hole transfer to the **PEH-9** molecule is an efficient pathway for the photogenerated charges. It is worth noting that due to the long absorption of **PEH-9** (Fig. 2), the excitation at 460 nm also excites, although to a minor extent, the HTM itself.

## Conclusions

We have synthesized three new donor–π–donor type hole transporting materials denoted as **PEH-3**, **PEH-8** and **PEH-9**, incorporating thiophene or thienothiophene with two electron-rich TPA units, *via* facile synthesis for perovskite solar cells. Suitable band alignments of the three molecules with perovskite and the photoanode allowed cascade electron injection and effective hole extraction in the perovskite solar cells. We compared and contrasted their optoelectronic properties and performance when acting as HTMs. The optimized devices of **PEH-9** exhibited an impressive PCE of 16.9% under standard global AM 1.5 illumination with minimized hysteretic behaviour, which is comparable to devices using a state-of-the-art spiro-OMeTAD hole transport layer under similar conditions. Single-crystal measurements and time-resolved photoluminescence (TRPL) revealed that the donor–π-bridge–donor structure with a planar configuration could be a promising strategy to design small molecule HTMs, due to the multiple intermolecular short contacts acting as charge transporting channels and efficient charge extraction properties from the perovskite layer. Ambient stability after 400 h revealed that 93% of the energy conversion efficiency was retained for **PEH-9**, similarly to the result for spiro-OMeTAD, indicating that the devices had good long-term stability. Studies on molecular engineering of donor–π–donor type HTMs that exhibit further increased charge transporting channels for highly efficient PSCs are now in progress.

## Author contributions

S. P., K. R. and P. G. conceived and designed the experiments, including synthesis and analysis of the data. S. P. and I. Z. fabricated PSC devices. P. G. refined the crystal data and analysed and did DFT calculation. G. G. measured and analysed static and transient photoluminescence. P. Gratia measured hole mobility. S. P. and P. G. wrote the first draft of the paper. All the authors contributed to the discussion and the writing of the paper, and approved. M. K. N. directed the scientific research for this work.

## Supplementary Material

Supplementary information

Crystal structure data
