# Supplementary material for: Donor–π–donor type hole transporting materials: marked π-bridge effects on optoelectronic properties, solid-state structure, and perovskite solar cell efficiency
Source: Chem Sci. 2016 May 24;7(9):6068–75. doi: 10.1039/c6sc01478j (PMC6022229; doi:10.1039/c6sc01478j)
Supplement: Supplementary file 1 [file SC-007-C6SC01478J-s001.pdf]

Electronic Supplementary Material (ESI) for Chemical Science.
This journal is © The Royal Society of Chemistry 2016

Supporting Information

Donor-π-Donor Type Hole Transporting Material: Marked π-Bridge Effects on Optoelectronic Properties, Solid-State Structure, and Perovskite Solar Cell Efficiency

S. Paek,[a] I. Zimmermann,[a] P. Gao,*[,a] P. Gratia,[a] K. Rakstys,[a] G. Grancini,[a] Md. K. Nazeeruddin*[,a]

[a] Group for Molecular Engineering of Functional Materials, Ecole Polytechnique Federale de Lausanne Valais Wallis, Rue de l'Indutrie 17, 1950 Sion, Valais, Switzerland.

Malik Abdul Rub,[b] Samia A. Kosa,[b] Khalid A. Alamry,[b] Abdullah M. Asiri,[b]

[b] Center of Excellence for Advanced Materials Research (CEAMR), King Abdulaziz, University, Jeddah, Saudi Arabia.

E-mail: mdkhaja.nazeeruddin@epfl.ch; peng.gao@epfl.ch

General Methods. All reactions were carried out under a nitrogen atmosphere. All reagents were purchased from Sigma-Aldrich, TCI and Alfa Aesar. ^{1}H and ^{13}C NMR spectra were recorded on a Bruker 400 MHz AVIII HD spectrometer. Mass spectra were recorded on a JEOL JMS-SX102A instrument. The absorption and photoluminescence spectra were recorded on a Perkin-Elmer Lambda 950S UV/VIS/NIR spectrometer and a Perkin LS-55 fluorescence spectrometer, respectively.

Electrochemical characterization. Cyclic voltammetry and differential pulse voltammetry were carried out with a SP-200 (BioLogie Company). A three electrode system was used and consisted of glassy carbon working electrode, platinum (diam. 1.0 mm, 99.9 % trace metals basis, Sigma-Aldrich) wire counter and reference electrodes were used with ferrocene as an internal standard. Redox potential of dyes was measured in CH_2Cl_2 with 0.1 M $(n\text{-}C_4H_9)_4N$-PF_6 as a scan rate of 50 mV s^{-1} (vs. Fc/Fc^+ as an external reference).

Cell fabrication. Conductive FTO glass (NSG10) was sequentially cleaned by sonication in a 2 % Helmanex solution and isopropanol for 15 min each, followed by a 10 min UV-ozone treatment. A 30 nm titania blocking layer was applied on the substrates by spraying a solution of titanium diisopropoxide bis(acetylacetonate) in ethanol at 450 degree. For the 150 nm mesoporous TiO_2 layer, 30 NR-D titania paste from Dyesol diluted in ethanol (150mg/ml) was applied by spin-coating at 4000 rpm for 10s followed by a sintering step at 450 degree for 30 min. The perovskite layers were fabricated by a single step spin-coating procedure reported by Soek et al. For the perovskite precursor solution 507 mg of PbI_2 (1.1mmol) and 159 mg (1mmol) of CH_3NH_3I were dissolved in 800 μl DMSO. The perovskite solution was spun at 4000 rpm for 30s using a ramp of 2000rpms^{-1}. 10s prior to the end of the spin-coating sequence 100 μl chlorobenzene were poured onto the spinning substrate. Afterwards the substrates were transferred onto a heating plate and annealed at 100 degree for 1h. The hole-transporting

materials were applied from a 50mM solution in tetrachloroethane for **PEH-9** and from a 20 mM solution in chlorobenzene for **PEH-3** and **PEH-8** respectively. Tert-butylpyridine (Tbp), Tris(2-(1H-pyrazol-1-yl)-4-tert-butylpyridine)cobalt(III) (FK209) and Tris(bis(trifluoromethylsulfon-yl)imide) (Li-TFSI) were added as additives. Equimolar amounts of additives were added for all hole-transporters: 330 mol% Tbp, 50 mol% Li-TFSI from a 1.8M stock solution in acetonitrile and 3 mol% FK209 from a 0.25M stock solution in acetonitrile. The final HTM solutions were spin-coated onto the perovskite layers at 4000rpm for 20s. The gold electrodes were deposited by thermal evaporation of 100 nm gold in high vacuum conditions.

Solar cell characterization. Current-voltage curves were measured in air under AM 1.5 simulated sunlight with a potentiostat (Keithley). The light intensity was calibrated with an NREL certified KG5 filtered Si reference diode. The solar cells were masked with a metal aperture of 0.16 cm^2 to define the active area. The current-voltage curves were recorded scanning at 10 mV s^{-1}.

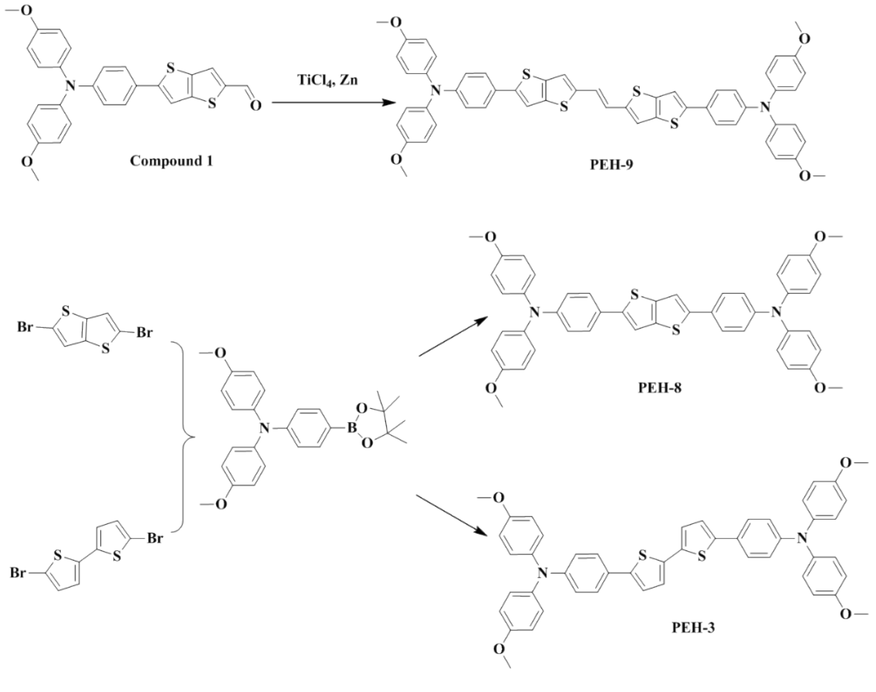

Scheme S1: Schematic diagram for the synthesis of three compounds

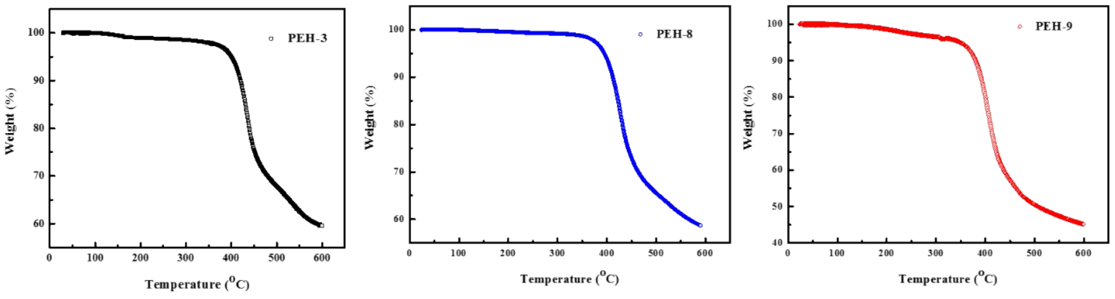

Figure S1. Thermal gravimetric analysis (TGA) measurement of **PEH-3**, **PEH-8** and **PEH-9**. Thermogravimetry analysis (TGA) showed that **PEH-3, PEH-8 and PEH-9** start to decompose at temperature around 410 °C, 400 °C and 370 °C, respectively.

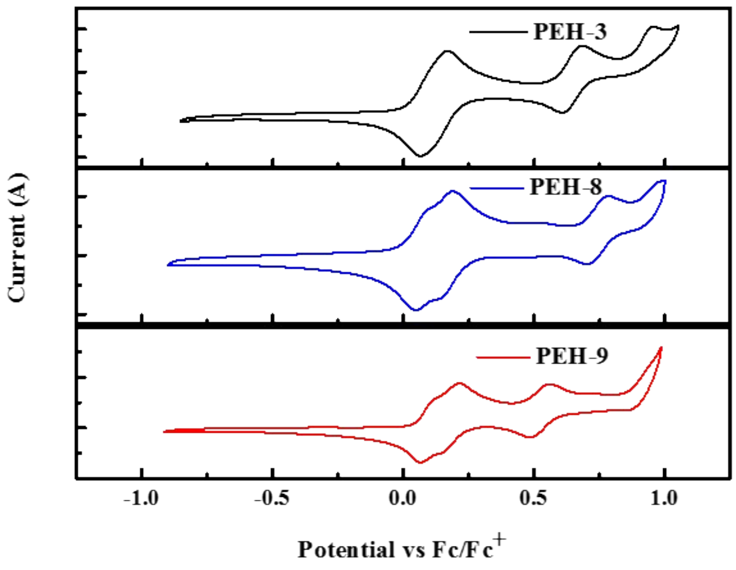

Figure S2. Cyclic voltammetry (CV) of the three compounds 0.1 M tetrabutylammonium hexafluorophosphate in CH_2Cl_2 at scan speed 50 mV/s, potentials vs. Fc/Fc^+

Computational methods

The geometry optimizations for the ground and charged states were performed by density functional theory (DFT) at the B3LYP/6-31G* level. The frontier molecular orbitals of the molecules for the PEH derivatives were calculated with an isovalue of 0.025 obtained by

DFT at the B3LYP/6-31G* level. All the DFT calculations were carried out with the Gaussian 09 package 2.

Hole Mobility Measurement

For the hole mobility measurement, solutions of HTMs with molar concentration of 70mM in chlorobenzene was prepared (doped with 3 mol % FK-209). The solution was spin-coated onto the OFET substrate (Fraunhofer IPMS) at 4000 rpm for 20 s. The mobility measurement was done on a 2.5μm channel (by length). The channel width was 10mm and the channel height was 40nm. The data were recorded with a Keithley 2612A by sweeping from -10 to 10 V (Source-drain voltage) at a scan rate of 1 V s^{-1}.

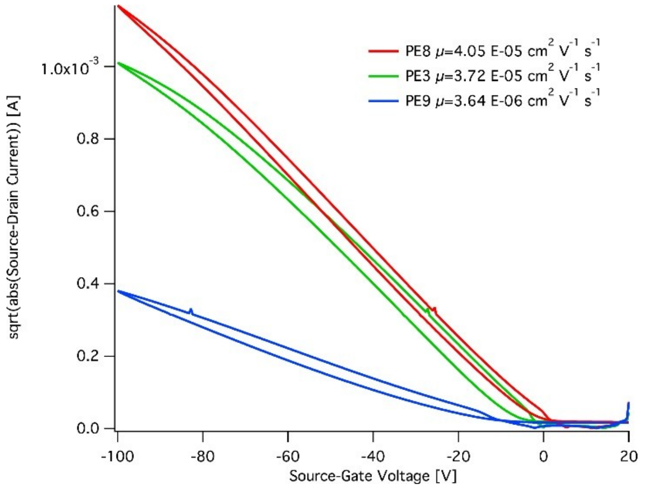

Figure S3. Mobility measurements on OFET substrates of the HTMs.

X-ray crystallographic analysis

The diffraction data of PEH series were measured at low temperature [100(2) K] using Mo Kα radiation on a Bruker APEX II CCD diffractometer equipped with a kappa geometry goniometer. The dataset was reduced by Eval*CCD* and then corrected for absorption. The crystal structure was solved and refined by *SHELX*. The crystal structure was refined using full-matrix least-squares based on F^2 with all non-hydrogen atoms anisotropically defined. Hydrogen atoms were placed in calculated positions by means of the

“riding” model.

Pseudo merohedral twinning was found in the last stages of refinement and treated by the TWINROTMAT algorithm of *PLATON*, obtaining one BASF value: 0.511(5). A summary of the crystallographic data, the data collection parameters, and the refinement parameters are given in Table S1. Crystallographic data have been deposited with the Cambridge Crystallographic Data Centre as supplementary publication numbers. These data can be obtained free of charge from the Cambridge Crystallographic Data Centre via www.ccdc.cam.ac.uk/data_request/cif.

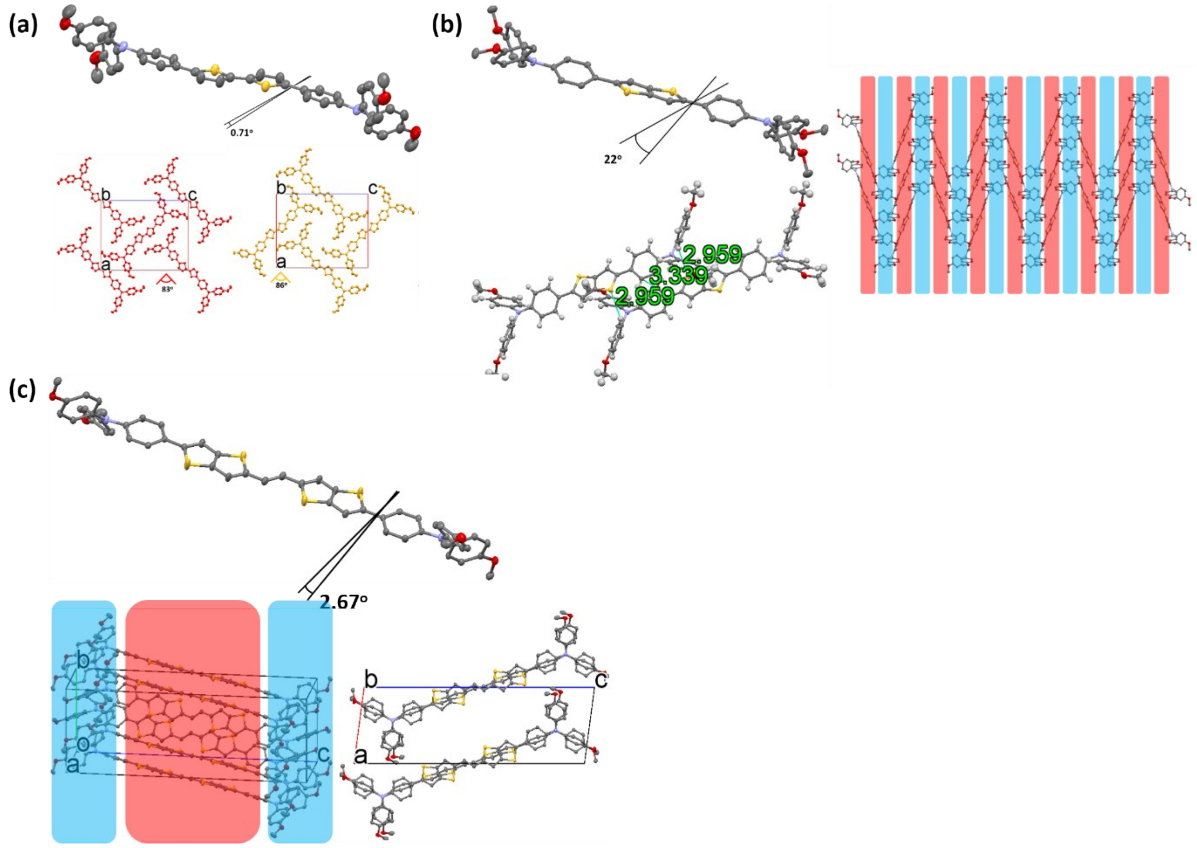

Figure S4. Crystal packings tructures and the dihedral angles between the triphenyl amine and center π system of (a) PEH-3; (b) PEH-8; (c) PEH-9. The black lines mark out the dihedral angles. The red, yellow, purple, and grey coloured atoms represent O, S, N, and C respectively (for clarity, the hydrogens are omitted).

Table S1. Crystallographic Data of All the new HTMs.

Compound Reference	***PEH-3***	***PEH-8***	***PEH-9***
Empirical formula	$C_{48}H_{40}N_2O_4S_2$	$C_{46}H_{38}N_2O_4S_2$	$C_{54}H_{42}N_2O_4S_4$
Formula weight	771	745	910.128
Wavelength (Å)	0.71073 (Mo Ka)	0.71073 (Mo Ka)	0.71073 (Mo Ka)
Crystal system	orthorhombic	monoclinic	triclinic
Space group	P b c a (61)	P 1 21/n 1 (14)	P -1 (2)
a (Å)	19.9041	11.0264	10.0156
b (Å)	8.9529	10.3433	10.1116
c (Å)	24.9415	17.0214	29.1958
α (°)	90.000	90.000	92.4450
β (°)	90.000	93.313	96.7000
γ (°)	90.000	90.000	104.7350
Unit cell volume ($Å^3$)	4444.56	1938.037	2831.93
Density (calc) ($g\ cm^{-3}$)	1.155	1.28	1.561
Temperature (K)	293 K	120 K	140 K
No. of formula units per unit cell	4	2	3
Completeness to theta	0.998	0.986	0.855
Largest diff. peak and hole ($Å^{-3}$)	0.613 and -0.215	0.385 and -0.352	0.408 and -0.594
No. of reflections measured	3910	5567	13349
No. of independent reflections	2110	4221	8124
Rint	0.099	0.038	0.054
GooF	1.153	1.164	1.048
Final R1 values (I > 2s(I))	0.0763	0.0525	0.686
Final wR(F2) values (I > 2 s (I))	0.02315	0.1335	0.1949
CCDC	1446683	1446684	1446682

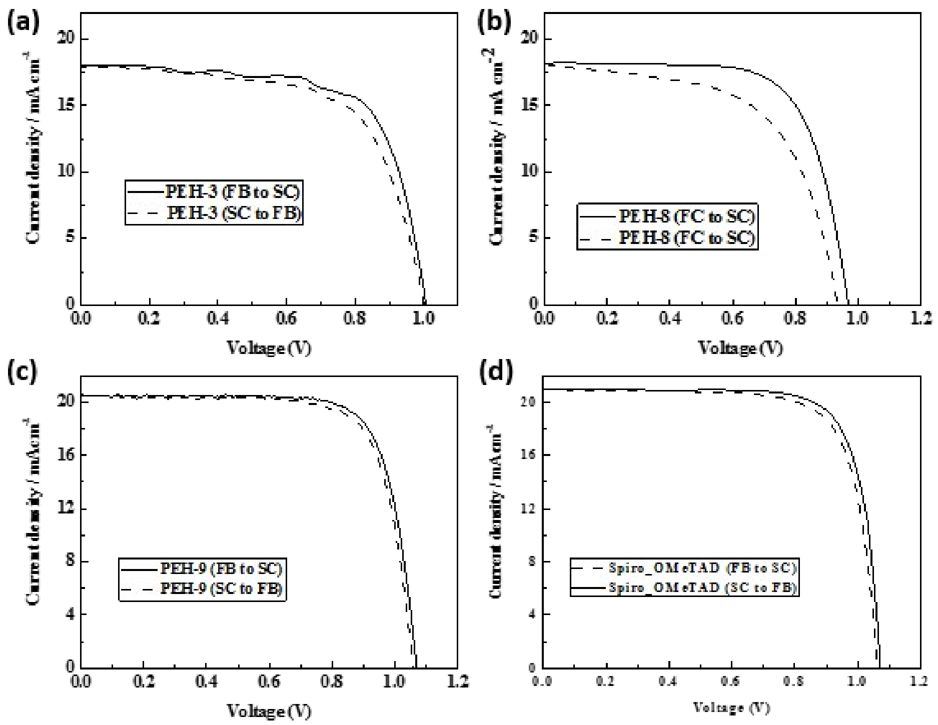

Figure S5. Current (J)-voltage (V) curves of the solar cells collected under AM1.5 simulated sun light. The curves were recorded scanning at 0.01 V s^{-1} from forward bias (FB) to short circuit condition (SC) and the other way round.

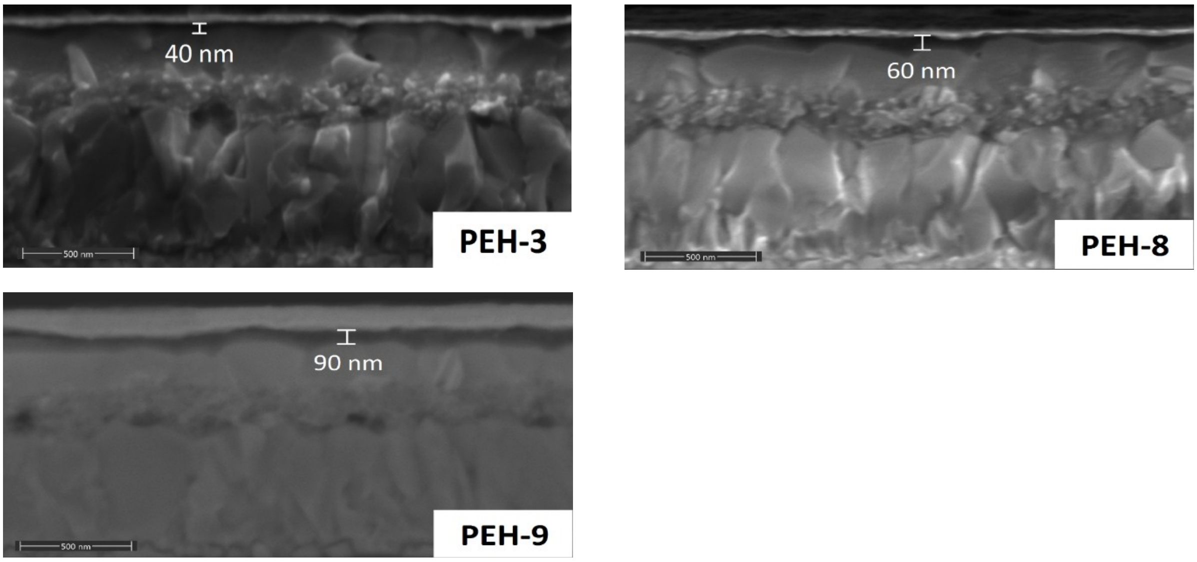

Figure S6. Scanning electron microscopy (SEM) picture of the cross section of the mp-TiO_2/$MAPbI_3$/HTMs/Au.

Photoluminescence Experiment

The time-resolved PL experiments were performed with a spectrophotometer (Gilden Photonics) using a pulsed source at 460 nm (Ps diode lasers BDS-SM, pulse with < 100 ps, from Photonic Solutions, approx. 1 mW power, 20 MHz repetition rate, approx. 500 um spot radius) and the signal was recorded at 760 nm by the Time Correlated Single Photon Counting detection technique with a time resolution of 1 ns. The samples were encapsulated to prevent any oxygen effects; the measurements have been performed under ambient conditions. A monoexponential and bi-exponential fitting were used to analyze the PL decay signal.

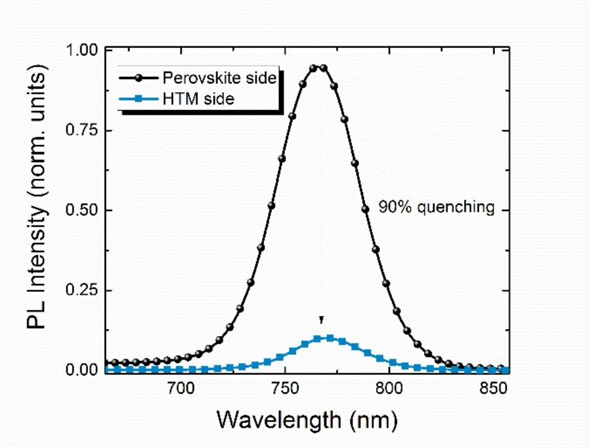

Figure S7. Continuous wave (CW) photoluminescence spectra of PEH9 exciting from the perovskite side and from the HTM side demonstrating a 90% quenching, excitation at 500 nm. All the samples have been encapsulated to prevent degradation or any oxygen/moisture induced effects.

Table S2. fitting parameters in terms of amplitude and time constant for the PL dynamics.

	A_1 (%)	***A_2 (%)***	***t_1 (ns)***	***t_2 (ns)***
Perovskite	100%		31	
spiro	88%	12	2.6	28
PEH3	85	15	1	15.5
PEH8	50	50	1	13
PEH9	98	2	1	12

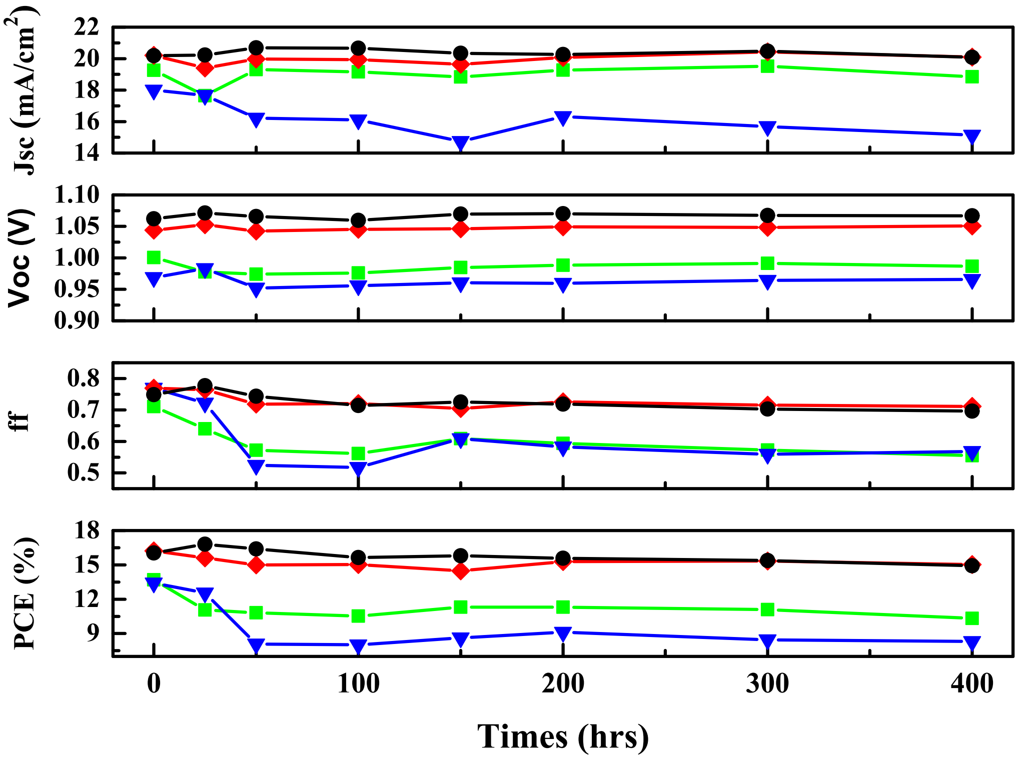

Figure **S8**. Stability test for devices based on **PEH-3** (■), **PEH-8** (▼), **PEH-9** (◆), and **Spiro** (●).

Table S3. A summary of stability data

	Times	Voc	Jsc	ff	H
PEH-3	0 h	1.0005	19.2575	0.711	13.6989
	25 h	0.977667	17.64333	0.64	11.05333
	50 h	0.974	19.29867	0.572	10.81667
	100 h	0.976	19.15533	0.561667	10.52333
	150 h	0.984667	18.83567	0.609	11.30333
	200 h	0.988333	19.267	0.594	11.30667
	300 h	0.991333	19.51633	0.572667	11.08333
	400 h	0.986667	18.84767	0.555	10.33
PEH-8	Times	Voc	Jsc	ff	η
	0 h	0.968667	17.995	0.769	13.42333
	25 h	0.983	17.66233	0.722	12.55

	50 h	0.952	16.21467	0.524	8.09
	100 h	0.955667	16.11	0.517333	8.01
	150 h	0.960333	14.734	0.608667	8.616667
	200 h	0.959667	16.31933	0.582667	9.116667
	300 h	0.964333	15.688	0.559	8.46
	400 h	0.965667	15.13567	0.568	8.3
PEH-9	Times	Voc	Jsc	ff	η
	0 h	1.044	20.207	0.769333	16.22333
	25 h	1.052667	19.40367	0.764	15.60667
	50 h	1.042667	19.975	0.718667	14.99
	100 h	1.045333	19.94333	0.72	15.02333
	150 h	1.046333	19.63667	0.704333	14.48
	200 h	1.049333	20.06733	0.725667	15.29
	300 h	1.048333	20.42933	0.715333	15.33
	400 h	1.050667	20.09467	0.711333	15.03
Spiro	Times	Voc	Jsc	ff	η
	0 h	1.062	20.1855	0.7485	16.045
	25 h	1.07125	20.2255	0.77675	16.8125
	50 h	1.06575	20.688	0.74375	16.385
	100 h	1.0595	20.66825	0.71425	15.635
	150 h	1.06975	20.34025	0.72525	15.7925
	200 h	1.07025	20.26025	0.7185	15.58
	300 h	1.0675	20.473	0.703	15.3825
	400 h	1.06675	20.0785	0.696	14.9225

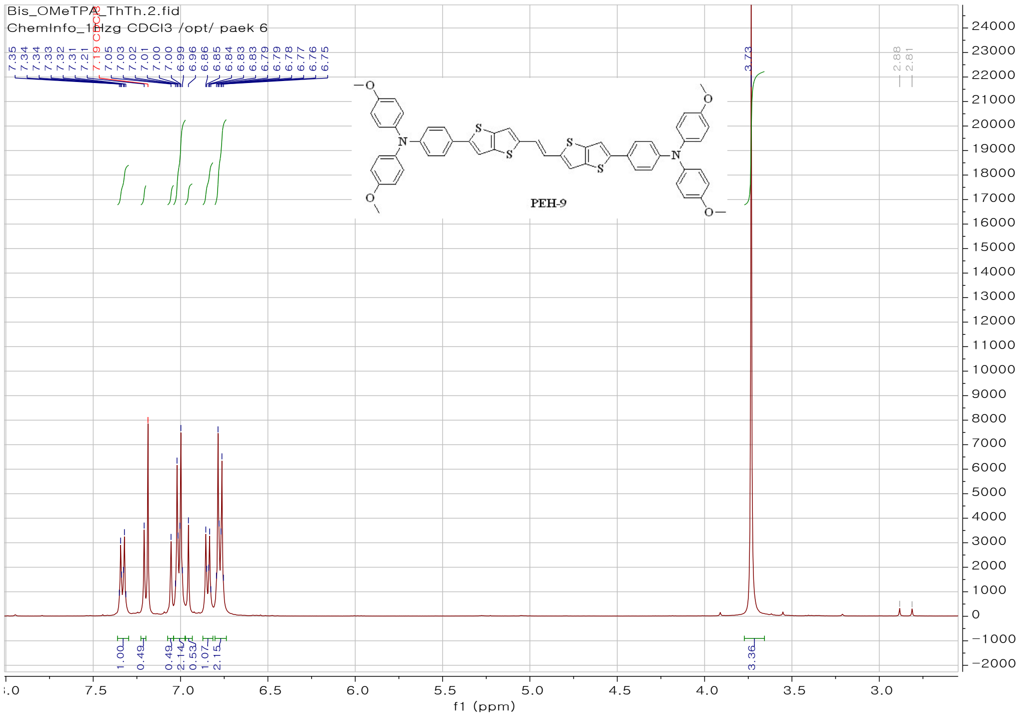

Figure S9. ^{1}H-NMR of **PEH-9**.

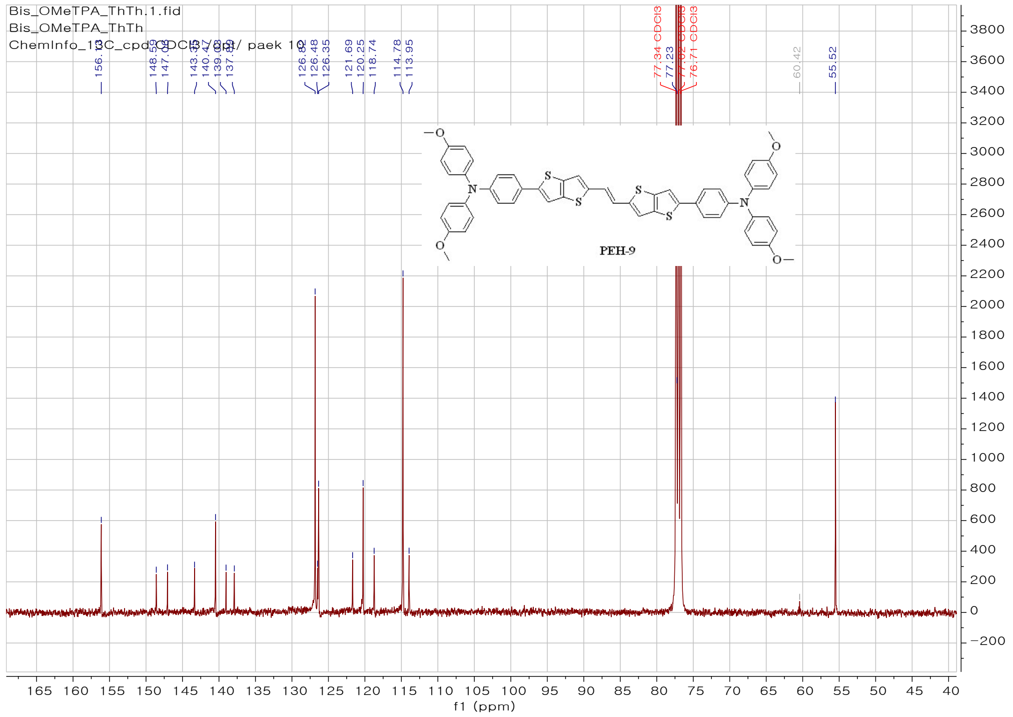

Figure S10. ^{13}C-NMR of **PEH-9**.

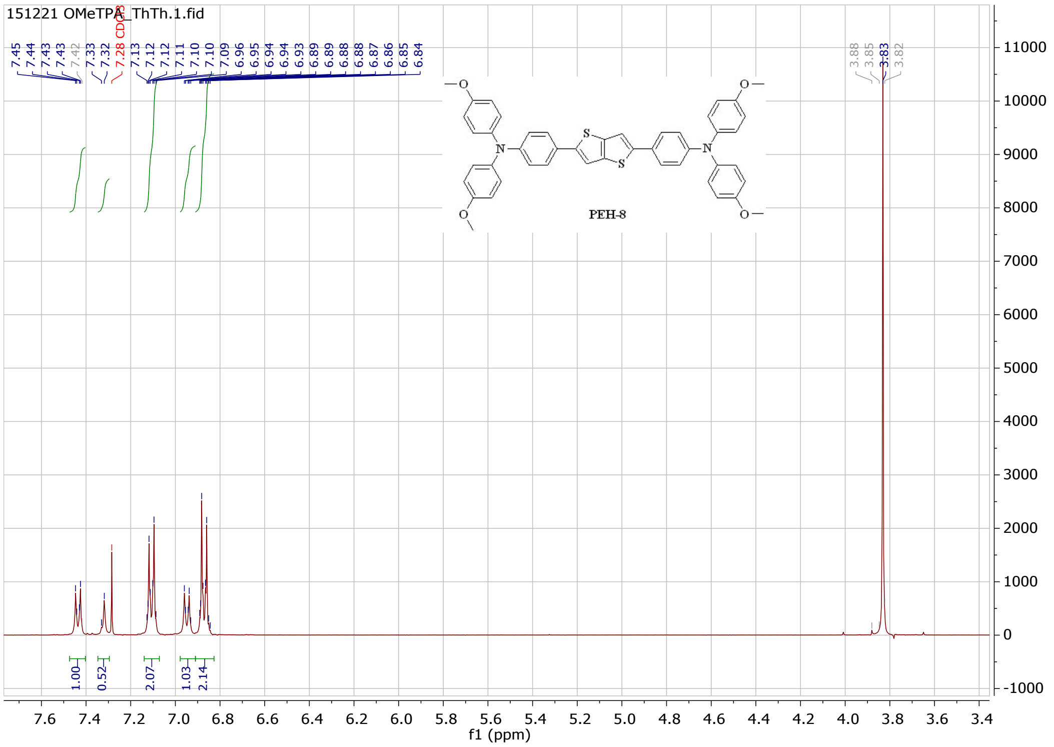

Figure S11. ^{1}H-NMR of **PEH-8**.

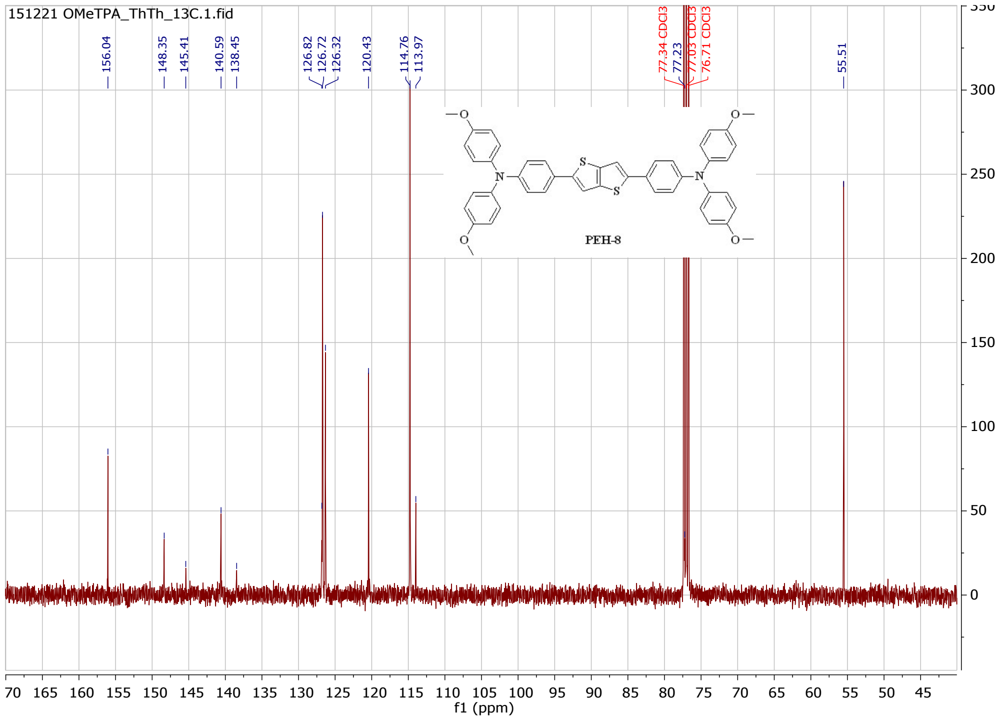

Figure S12. ^{13}C-NMR of **PEH-8**.

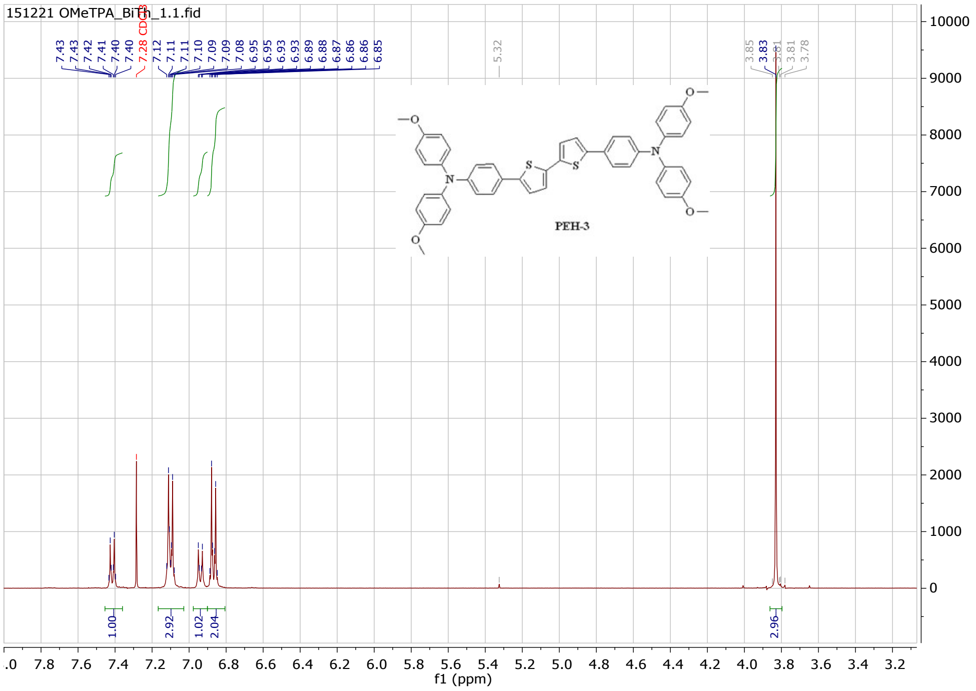

Figure S13. ^{1}H-NMR of **PEH-3**.

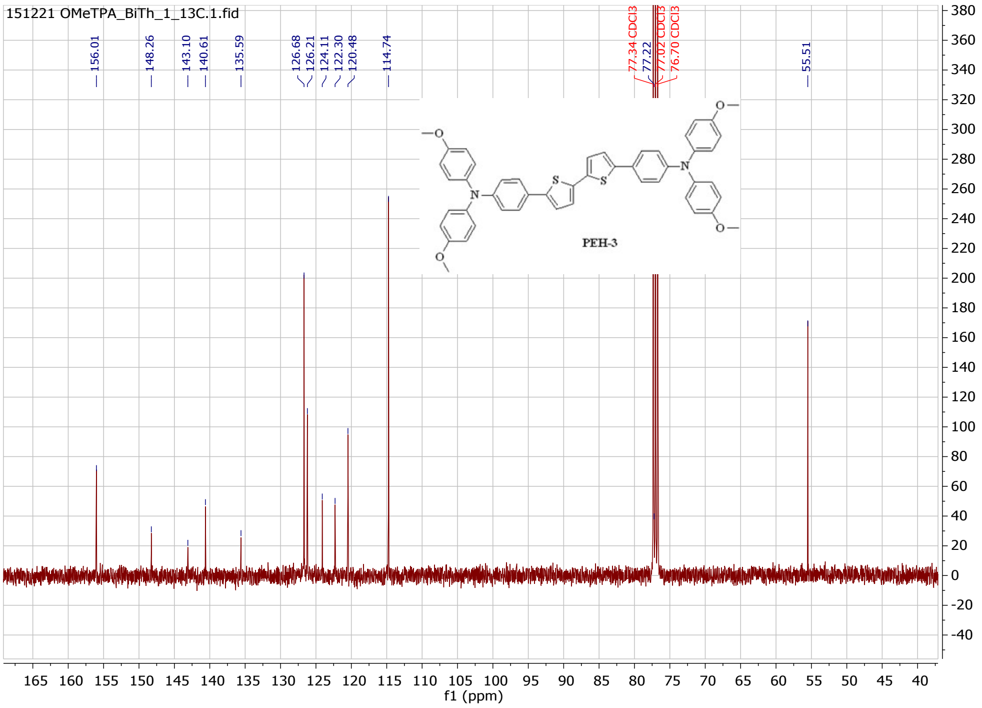

Figure S14. ^{13}C-NMR of **PEH-3**.
